# Going beyond ‘regular and casual’: development of a classification of sexual partner types to enhance partner notification for STIs

**DOI:** 10.1136/sextrans-2020-054846

**Published:** 2021-04-29

**Authors:** Claudia S Estcourt, Paul Flowers, Jackie A Cassell, Maria Pothoulaki, Gabriele Vojt, Fiona Mapp, Melvina Woode-Owusu, Nicola Low, John Saunders, Merle Symonds, Alison Howarth, Sonali Wayal, Rak Nandwani, Susie Brice, Alex Comer, Anne M Johnson, Catherine H Mercer

**Affiliations:** 1 School of Health and Life Sciences, Glasgow Caledonian University, Glasgow, UK; 2 Sandyford Sexual Health Service, NHS Greater Glasgow and Clyde, Glasgow, UK; 3 School of Psychological Sciences and Health, University of Strathclyde, Glasgow, UK; 4 Primary Care and Public Health, Brighton and Sussex Medical School, Brighton, Brighton and Hove, UK; 5 Institute for Global Health, University College London, London, UK; 6 Institute of Social and Preventive Medicine, University of Bern, Bern, Switzerland; 7 Blood Safety, Hepatitis, STI & HIV Division, Public Health England, London, UK; 8 Department of Sexual Health, West Sussex Health and Social Care NHS Trust, Worthing, West Sussex, UK; 9 Development Media International CIC, London, UK; 10 Barts Health NHS Trust, London, UK; 11 Central and North West London NHS Foundation Trust, London, UK; 12 Department of Infection & Population Health, University College London, London, UK; 13 Centre for Sexual Health and HIV Research, University College London, London, UK

**Keywords:** sexual health, contact tracing, chlamydia infections, gonorrhea, HIV

## Abstract

**Objectives:**

To develop a classification of sexual partner types for use in partner notification (PN) for STIs.

**Methods:**

A four-step process: (1) an iterative synthesis of five sources of evidence: scoping review of social and health sciences literature on partner types; analysis of relationship types in dating apps; systematic review of PN intervention content; and review of PN guidelines; qualitative interviews with public, patients and health professionals to generate an initial comprehensive classification; (2) multidisciplinary clinical expert consultation to revise the classification; (3) piloting of the revised classification in sexual health clinics during a randomised controlled trial of PN; (4) application of the Theoretical Domains Framework (TDF) to identify index patients’ willingness to engage in PN for each partner type.

**Results:**

Five main partner types emerged from the evidence synthesis and consultation: ‘established partner’, ‘new partner’, ‘occasional partner’, ‘one-off partner’ and ‘sex worker’. The types differed across several dimensions, including likely perceptions of sexual exclusivity, likelihood of sex reoccurring between index patient and sex partner. Sexual health professionals found the classification easy to operationalise. During the trial, they assigned all 3288 partners described by 2223 index patients to a category. The TDF analysis suggested that the partner types might be associated with different risks of STI reinfection, onward transmission and index patients’ engagement with PN.

**Conclusions:**

We developed an evidence-informed, useable classification of five sexual partner types to underpin PN practice and other STI prevention interventions. Analysis of biomedical, psychological and social factors that distinguish different partner types shows how each could warrant a tailored PN approach. This classification could facilitate the use of partner-centred outcomes. Additional studies are needed to determine the utility of the classification to improve measurement of the impact of PN strategies and help focus resources.

## Introduction

Understanding the nature and number of sexual partners of people with STIs is fundamental to understanding the epidemiology of STIs, delivery of high-quality clinical care and prevention of transmission through effective partner notification (PN).^1–6^ However, we need appropriate tools to assess to whom and how interventions should be targeted.

Specialist sexual and public health guidance and published researchers tend to use a simple dichotomy of ‘regular’ (‘steady’) or ‘casual’ sexual partners.^3 7–10^ These categories do not take into account the complexities of sexual partnerships in ways that help understand the potential for STI transmission or support clinical, research or prevention practice. The outcomes of PN generally specify an overall number of partners contacted/treated per index patient,^3^ ignoring variation in the timing and types of partnerships, the likelihood of onward transmission by partnership type^1^ or the different kinds of support needed to notify partners effectively.^6^

The way people meet sex partners is changing. Through increasing internet use,^11^ online commercial socio/sexual networking sites have generated their own partner classifications, shaping the ways people understand and talk about relationships.^12^ Public awareness of different types of sexual partners is also increasing, with recognition of sexual interactions where the label of ‘partner’ is not applicable. These changes are taking place at a time of sustained rises in STIs in some groups.^13^

Social epidemiologists and behavioural scientists have sought to develop alternative ways of classifying partnership type to try and better understand STI and HIV risk (eg, refs 14–16), but there is no consensus. As a result, we lack comparable quantitative data for epidemiological studies and service evaluations. A standardised partner type classification, with face validity for both patients and service providers, would improve measurement of the impact of PN strategies and help focus resources.^17^ If applied to the practice of PN, a new classification would help a move towards *partner*-centred outcomes (eg, transmissions prevented according to partnership type) rather than *patient-*centred outcomes (eg, partners tested/treated per index case).

The objectives of this study were to create a useable classification of sexual partners to improve the targeting of PN for STIs. The study addressed four questions: (1) which labels are used to classify sexual partners and which biomedical, psychological and social aspects differentiate them?; (2) what does a classification of sexual partners look like for clinical practice?; (3) is this classification acceptable and feasible for routine use?; and (4) how could use of the classification help to improve individual and public health?

## Methods

This study is part of the Limiting Undetected Sexually Transmitted infections to RedUce Morbidity (LUSTRUM; lustrum.org.uk) research programme. We used mixed research methods within a broad, biopsychosocial approach, acknowledging the importance of psychological, social and cultural factors to the understanding of sexual partnerships.^18^ We used a four-step process: (1) integrating evidence from diverse sources to develop an initial classification, (2) multidisciplinary clinical expert input to revise the classification, (3) piloting the classification in sexual health clinics and (4) application of the Theoretical Domains Framework (TDF)^19^ to explore the need for tailored PN.

### Evidence integration

We iteratively synthesised the findings from four diverse sources of evidence: (1) a scoping review of partnership types described in the social and health sciences literature,^20^ (2) a review of PN guidelines,^21^ (3) focus group discussions with lay people, including those recently diagnosed with an STI^22^ and (4) a review of partnership types described in dating apps.^12^

The methods used for each source have been published separately.^12 20–22^

We created a matrix of partner types, according to the key biomedical, psychological and social factors that differentiated them. First, we derived descriptions of partner types from the review of social and health sciences literature.^20^ Second, we used constant comparative techniques, that is, taking published data and comparing them with our emerging findings and putting ‘like with like’ to map descriptions of types of relationship and concomitant partner type from the other data sources (the reviews of dating apps, PN intervention content and guidelines and findings from focus group discussions). We applied the same approach to identify the key biomedical, psychological and social factors that differentiated the particular types of relationships and partners.

### Multidisciplinary clinical expert input

Experts provided opinions and suggestions in the following sequence: (1) we discussed initial drafts of the matrix with the full LUSTRUM project team, which includes clinical sexual health and PN specialists, (2) we sought opinions on a revised draft from multidisciplinary clinical sexual healthcare professionals in a workshop on PN outcomes at a national specialist conference (BASHH Annual Spring Meeting, 2017), (3) a senior team member with clinical expertise (CSE) applied the feedback from the workshop participants to examine the conceptual coherence and logic of the matrix. She assessed its utility against a range of real and hypothetical patient scenarios and discussed areas of uncertainty and disagreement with the LUSTRUM team. (4) We simplified the matrix again to improve clinical utility. This process produced the sex partner classification that the project team considered clinically useful; and (5) STI clinical, public health and epidemiology experts from UK, Australia and The Netherlands gave further input as part of a BASHH working group to develop PN outcomes in May 2019, and changes were incorporated.

### Piloting the classification

We developed a standardised 15 min training session for healthcare professionals about the refined classification and how to use it during sexual history taking and PN consultations. The training was part of a randomised controlled trial (RCT) of accelerated partner therapy for PN for heterosexual people with chlamydia.^23^ Around 120 healthcare professionals (nurses, health advisers and doctors) received the training between 31 March 2018 and 26 September 2018 at 17 sexual health clinics in England and Scotland, which were purposively selected for their contrasting patient populations and geographical contexts. At the end of the training, we administered an informal quiz using patient scenarios. Healthcare professionals practised using the classification and discussed answers collectively. Healthcare professionals used the classification during the pretrial baseline data collection phase and the first trial period (4 November 2018–28 April 2019). As part of the trial’s monthly clinic support sessions, we asked each clinic’s ‘trial champion’ about their team’s experiences using the classification for sexual histories and PN, specifically noting any instances where clinicians had felt unable to assign a particular patient’s partner to any category.

### Applying the TDF to the classification

To understand how the classification might enhance PN, we explored how, from an index patient’s perspective, relative barriers and facilitators to engaging with PN differed according to partnership type. We used the TDF to code these findings.^19^ In this way, we outlined the differential barriers and facilitators shaping index patient engagement with PN in order to speculate about how the classification might suggest particular tailored PN approaches for different partner types.

## Results

The results are presented in relation to the research questions.

### Which labels are used to classify sexual partners and which biomedical, psychological and social aspects differentiate them?

**Figure 1:**
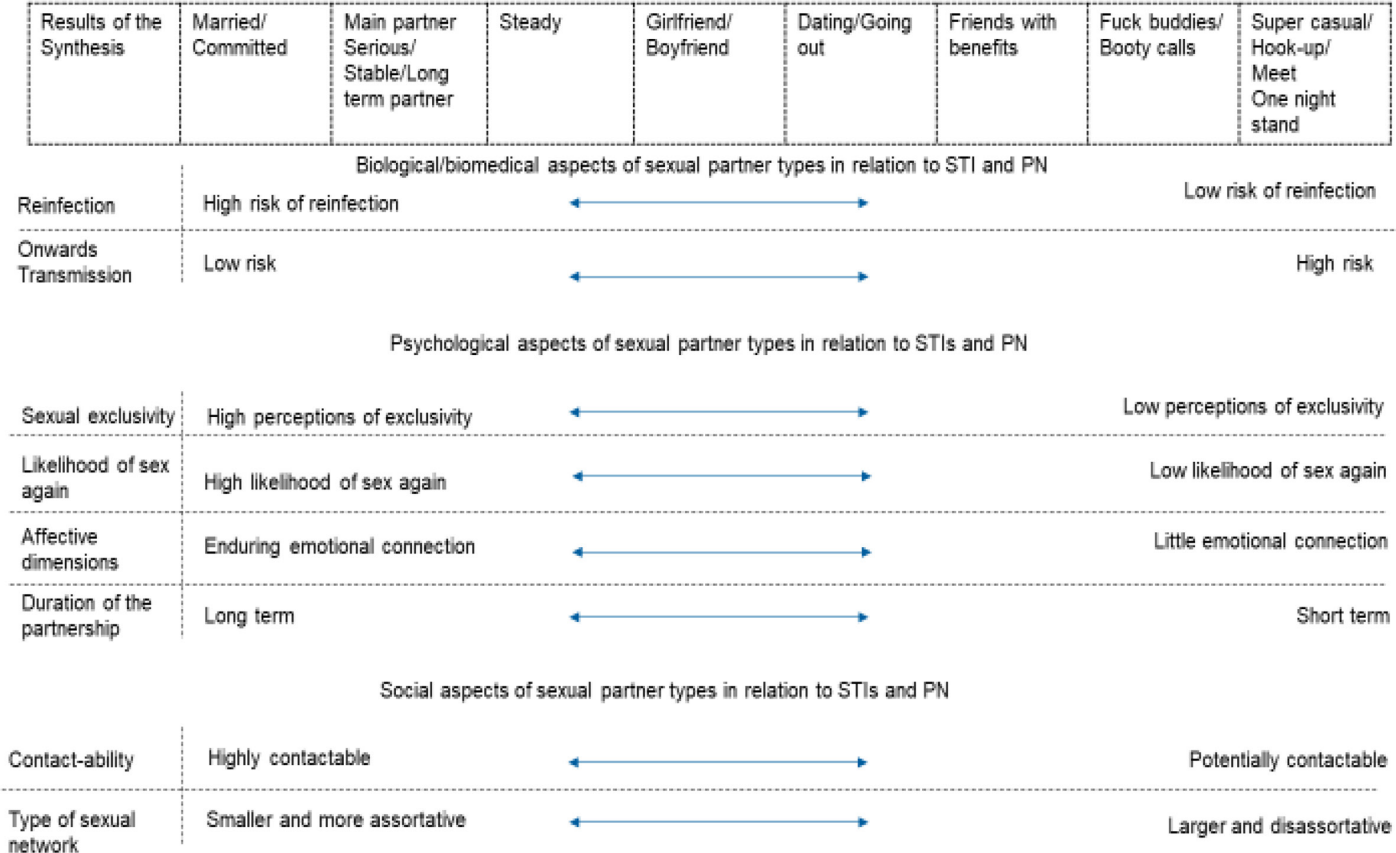
Initial matrix of sexual partner types and the key features that differentiate them. Evidence synthesis derived an initial eight partner types (top row). Factors that differentiated them are shown in the left-hand column.

The evidence integration initially resulted in eight categories, which summarised the range of identified sexual partner types (figure 1, top row). These types span (from left to right) the traditional dichotomy from ‘regular’ to ‘casual’. The partner types also incorporate trajectories across time, with intensity and duration decreasing from left to right. We identified eight important variables: two in the biomedical domain, four psychological factors and two social aspects, which could help distinguish between partnership types. The resulting matrix represents a visualisation of our initial best fit of partner types against varied biospsychosocial characteristics. It is not intended to provide a detailed veridical or testable model.

From a biomedical perspective, the classification captures the concept that both the likelihood of STI reinfection within the partnership *and* onwards transmission to other people differ by partner type.^1^ Reinfection within a partnership is more pertinent for partner types such as ‘married/committed’ and ‘main partner’/‘serious partner’/‘stable partner’/‘long term partner’, while onward transmission is more of a risk at the opposite end of the partner spectrum (‘super casual’/‘hook up’/‘meet’ and ‘one-night stand’).

We identified key psychological factors that appeared to differ between partner types: higher perceptions of an exclusive partnership, higher likelihood of sex again and more enduring emotional connection are associated with those types at the lefthand side (ie, towards ‘married’/‘committed’).

In contrast, types characterised by lower expectations of exclusivity, lower likelihood of sexual intercourse with that partner again, little emotional connection and shorter duration cluster on the righthand side towards the ‘super casual’/‘hook-up’/‘meet’/‘one-night stand’ partner types.

Social factors also distinguish between partner types. For example, partner types towards the right-hand side of figure 1 are more likely to be embedded within larger, disassortative, multifaceted sexual networks than those towards the left-hand side. Contactability is less clearly polarised and may be possible all across the partner spectrum but is likely to reduce from left to right.

### What does a classification of sexual partners look like for clinical practice?

**Figure 2:**
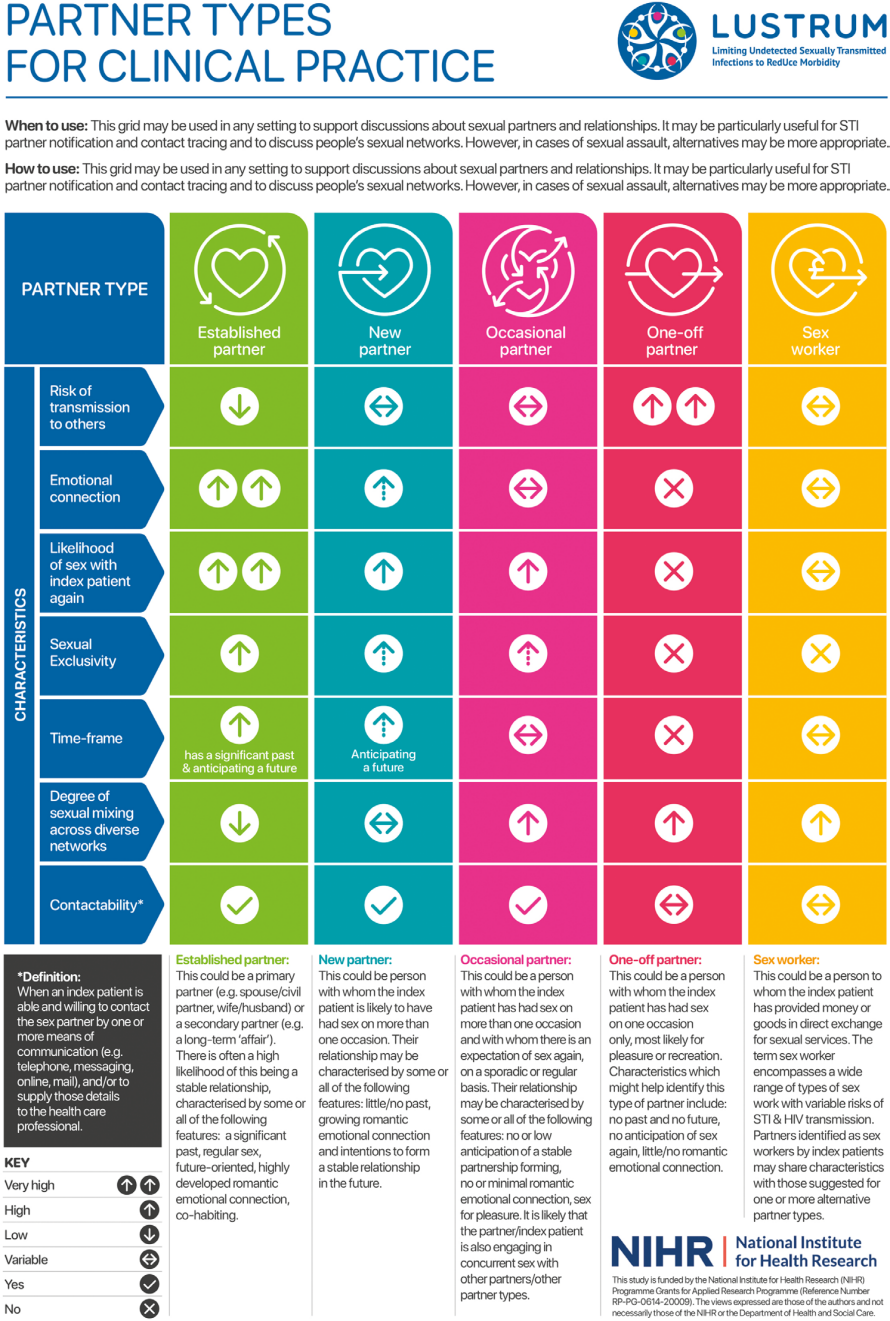
Sexual partner types for clinical practice.

We simplified the initial classification from eight to four categories to make it practical for use in clinical care based on the multidisciplinary clinical expert input (figure 2). The clinician researchers recommended adding a fifth category of sex partner ‘sex worker’, which had not emerged from the scoping review as a separate partnership type. A separate category for sex work reflects UK national guidance on sexual history taking^24^ and because PN and risk reduction strategies are likely to differ for this group. The research team assigned short neutral labels to each category as an aide-memoire for healthcare professionals. The labels are not intended to be used as descriptors in consultations with patients although some of the words used may figure in patients’ descriptions of their relationships, for example, ‘One-offs’ and ‘Sex worker’.

### Is the classification acceptable and feasible for use in routine clinical practice?

Informal verbal feedback from the pretrial teaching sessions was overwhelmingly positive. Participants agreed that the new categories could help overcome the well-recognised limitations of the ‘regular/casual’ dichotomy. Participants correctly assigned partners to categories in the post-training skills test. During the trial baseline data collection phase, we discussed and resolved categories for a small number of clinical cases, raised by clinical staff. There were no further queries after starting the trial, and by the end of the first trial period (4 November 2018 to 28 April 2019), clinicians across the 17 study sites had used the classification to categorise 3288 sex partners from 2223 index patients. There were no instances in which clinicians felt unable to assign a sex partner to any category. Figure 2 summarises the partner types for use by healthcare professionals in sexual health clinics.

### How could use of the classification help to improve individual and public health for PN?

Table 1 illustrates the ways that the different partner types within the classification may need different PN approaches.

**Table 1:** Use of Theoretical Domains Framework (TDF): implications of the classification of partner types for partner notification (PN) approaches

Partner type*	Established partner	New partner	Occasional partner	One-off partner
Selected Theoretical Domains Framework functions that discriminate index patient’s engagement in PN	Knowledge			
	Good knowledge of partner likely. This might facilitate PN; the index patient may anticipate their reaction and their respective choice of PN approach.	Uncertain knowledge of the partner, their reactions, their choice of PN approach.	Good knowledge of partner likely. This might facilitate PN; the index patient may anticipate their reaction and their respective choice of PN approach.	Very little of the partner may facilitate or constrain engagement in PN.
	**Social role and identity**			
	(Eg, spousal roles) May facilitate motivation to engage in PN according to scripts, expectations and assumptions.	(Eg, romantic roles) May constrain engagement in PN, although novelty of relationship ‘permits’ residual infections.	Scripts and norms (eg, ‘friends with benefits’) may enable engagement in PN.	Identities (eg, being a player) may constrain engagement in PN.
Implications for PN	*Beliefs about the consequences* of engaging in PN are important and varied (health, interpersonal and infidelity).	*Beliefs about the consequences* of engaging in PN are important and varied (health, interpersonal and end of the relationship).	*Beliefs about the consequences* of engaging in PN are probably health oriented and may facilitate PN.	Likely to have minimal concerns about the *consequences* of engaging in PN.
	*Emotions* may be particularly important given length of relationship and likely expectations of exclusivity.	*Emotions* may be particularly important given potential emerging expectations of exclusivity; they may be particularly intense.	*Emotions* may be less important and not represent barriers to engaging in PN.	*Emotions* are not likely to be important in relation to engaging in PN.
	Highly amenable to PN interventions that use the existing relationship dynamics, rapid effective access to partners, established routes of communication and almost guaranteed future interactions between partners.	PN interventions that rely on existing relationship dynamics may be problematic and access to partners may be difficult.	Highly amenable to PN interventions that can use the existing relationship dynamics, established means of access to partners, established routes of communication and almost guaranteed future interactions between partners.	PN for these types of partner maybe more resource intensive as index patients may have low motivation to engage in PN.
	Depending on the nature of the particular relationship, there may be emotional issues that may delay or prolong PN. Additional support and services may be warranted.	Depending on the nature of the particular relationship, there may be emotional issues that delay, or constrain, some approaches to PN. These may require additional support and services.	There may be high motivation to engage in PN, and relatively few resources may be needed.	Patient referral may be challenging and may miss important public health opportunities.
		Framing effective PN as a commitment to the new relationship may be a beneficial approach.	PN approaches that motivate people to engage in PN because of health consequences and to protect their partner and themselves from future harms may be effective.	PN approaches that seek to motivate index patients through appeals to social norms and ideas of the social good may be effective. PN approaches that use the likelihood of reinfection and the health consequences to self are unlikely to work.
	PN approaches that rely on interpersonal dynamics (including accelerated partner therapy (APT)/expedited partner therapy (EPT)) are likely to work, although emotional aspects may be key barriers.	Approaches such as APT or wider EPT may be appropriate for some of these partners.	Approaches such as APT or wider EPT may be highly appropriate for these partners.	PN interventions that rely on interpersonal dynamics (including APT/EPT) are unlikely to work.
				May be particularly amenable to enhanced provider referral that engages fully with the mechanisms by which these partners met (eg, dating apps).

*The sex worker partner type was added to the classification after completion of this phase of work and so was not included in the TDF analysis.

For any index patient with multiple sexual partners, the TDF^19^ suggests it may be worth exploring which type(s) of sexual partner(s) they have and, subsequently, which type(s) of PN may be most appropriate for each different partner, depending on their type. Critically, although an index patient may have equal physical capability to engage in PN with diverse types of partner, there are important differences in the index patient’s motivation to engage in PN with different partner types. For example, from an index patient’s perspective, there may be very little motivation to engage in PN with ‘one-off partners’ and far more to engage with ‘established partners’ with whom sex is likely to occur again.

For ‘established partners’, PN approaches could benefit from using the deep emotional connection between partners, the likelihood of cohabitation and the high potential for reinfection. Approaches such as expedited partner therapy^25^ and accelerated partner therapy^23^ are likely to be acceptable and effective. Depending on the particular relationships and expectations concerning exclusivity, PN may be taking place against a background of high emotions and potential distress; partner delivered testing/treatment options may be very useful.

For ‘new partners’, PN approaches could benefit from harnessing the developing emotions within such relationships and capitalise on the relationship’s short duration. For these relationships, a diagnosis of an STI can ‘make or break’ the developing relationship. For example, it may be that the STI has arisen from sex pre-dating the current ‘new’ relationship, or that the STI has been transmitted before agreements concerning exclusivity have been made.

For ‘occasional partners’ characterised by high likelihood of the relationship, having a future and likely sex again, yet limited emotions, approaches such as expedited partner therapy,^25^ and accelerated partner therapy^23^ may be highly acceptable.

For ‘one-off partners’, PN approaches that require an emotional connection between partners, or those that use risks of reinfection to motivate partners or are unlikely to be effective. However, given changes in the ways people meet and the widespread use of social media, index patients may well have some means of contacting these types of sexual partner. Provider referral, in which the healthcare professional contacts the sex partner without revealing the identity of the index patient, may be useful.

## Discussion

We developed a new five-category classification of sex partner types. We synthesised diverse sources of evidence to understand the biomedical, psychological and social aspects that make the partner types identified distinct. The classification was feasible and acceptable to a range of healthcare professionals within sexual health services across England and Scotland. The classification accommodates most sex partner types described by people attending UK sexual health services, and staff were able to assign all sex partners described to a category.

For use in routine clinical care, a classification needs to be pragmatic, such that the majority of partner types described in contemporary life and clinical practice can be assigned to a reasonable number of categories, while recognising that some patients will describe partners who cannot be neatly assigned to any category. Our proposed classification goes beyond the mutually exclusive dichotomy of ‘regular’ or ‘casual’ partnerships that has been used in sexual health practice to date. By synthesising diverse sources of evidence, our classification considers the realities and increasing complexities of the contemporary social organisation of sexual relationships.

This work has drawn on, and further developed, existing classifications that typically focus on a single dataset and/or consider fewer partnership-specific variables to differentiate between the types identified. The classification has important differences from an earlier classification that was based on responses to questions in the third British National Survey of Sexual Attitudes and Lifestyles (Natsal-3).^16^ Questions in Natsal-3 distinguished between partnerships that involved cohabiting and those that were considered as ‘now steady’. We now propose using the collective label of ‘Established’. While the earlier classification had just one category for ‘casual’ partners, we now propose two categories: ‘occasional’ partners and ‘one-off’ partners. This distinction is helpful because of the greater heterogeneity in casual sex (and the labels attributed to this) as compared with more established partnerships. The distinction is also relevant to the delivery of PN, reflecting variation in the extent that different types of casual partners can be traced and/or contacted.

The partner types that emerged are culturally embedded in UK sexual health settings. Although we searched the international literature, the classification might not be generalisable to very different populations or cultures. While we piloted the usability of the classification during a trial that included only people who have sex with opposite gender partners, the partner types also make sense for same sex partnerships and those that include trans/transgender and non-binary people. The classification takes account of current societal sexual behaviours and so may not be relevant if significant shifts in sexual behaviours occur.

A pragmatic, evidence-informed classification could enhance clinical practice and research study design. More appropriate targeting and tailoring of PN and other sexual health interventions should result in greater individual and public health benefits. While our classification prioritises utility within the clinical context rather than the general population, it is informed by published evidence and primary research undertaken with people in a variety of settings, including clinic attendees and lay people.

The classification could improve the ability of services to address the aims of PN at both individual patient level (prevention of reinfection) and public health level (transmission prevention) by ensuring that the best available evidence guides the choice of PN methods offered by services. Tailored PN approaches should enable more effective targeting of resources and audit that is meaningful in epidemiological terms, as well as relating to the individual index patient. This methodological advance will also enhance social epidemiology and the evidence provided by behavioural surveillance to facilitate development of patient-centred risk assessment tools. Such tools will enable robust comparisons of the transmission prevention outcomes of existing and novel PN approaches as well as index patient-centred outcomes. Collectively, these advances could improve patient care by ensuring that best-available evidence guides choice of PN methods offered by services. At the individual patient level, an awareness of the distinct aspects of each partner type could enable better tailoring of PN interventions offered by healthcare professionals and allow a more strategic approach to prevention of transmission. This offers considerable potential when PN is particularly important at both the individual and public health level, such as with cases of extensively drug-resistant pathogens.^26^

The content validity of the classification is being evaluated in the RCT of accelerated partner therapy,^23^ which will include analysis of trial outcomes by partner type. Evaluation in clinical practice through a UK national audit will establish whether the classification accommodates most sex partner descriptions, including same sex partners, when embedded in routine care. Additional studies are needed to determine the utility of the classification to improve measurement of the impact of PN strategies and help focus resources. Future work will address tailored intervention development based on partner type, which could inform targeting of resources to reach sex partners who might contribute disproportionately to transmission within the population. New PN methods will need to embrace the range of communication technologies used within contemporary social and sexual networks and determine the cost-effectiveness of PN approaches with different types of partner in relation to reducing onwards transmission at the population level.

**Key messages**

- Current classifications of sexual partners limit understanding of STI transmission dynamics and hinder targeting and tailoring of partner notification interventions.
- The limits and constraints of current classifications, together with recent sociosexual changes, mean that a new classification is needed.
- We developed a comprehensive, evidence-based classification of sexual partner types for use in partner notification that characterised and distinguished between partner and partnership types.
- The five partner categories were readily adopted and easily operationalised in UK sexual health services.

10.1136/sextrans-2020-054846.supp1 Supplementary data

## Data Availability

Data are available on reasonable request from Professor Claudia Estcourt (corresponding author): claudia.estcourt@gcu.ac.uk. The data consist of anonymised qualitative interview and focus group data and may be used with permission for research and/or clinical purposes. More information about the LUSTRUM research programme is available on the study website www.lustrum.org.uk and in the trial protocol paper: doi:10.1136/bmjopen-2019-034806.
