# Supplementary material for: Going beyond ‘regular and casual’: development of a classification of sexual partner types to enhance partner notification for STIs
Source: Sex Transm Infect. 2021 Apr 29;98(2):108–14. doi: 10.1136/sextrans-2020-054846 (PMC8862076; doi:10.1136/sextrans-2020-054846)
Supplement: Supplementary data [file sextrans-2020-054846supp001.pdf]

Supplementary File:

Table S1: Towards a practical classification of partner types: Interim stage

<table>
<tr><th>Partner label/ name</th><th colspan="2">Established partner</th><th colspan="3">New Partner</th><th colspan="2">Occasional partner</th><th>One-off partner</th><th>Sex worker</th></tr>
<tr><td>Categories of partner from the initial matrix (Figure 1)</td><td>Married

Committed</td><td>Main Partner

Serious Partner

Stable Long term</td><td>Steady</td><td>Girlfriend

Boyfriend</td><td>Dating

Going Out</td><td>Friends with benefits</td><td>Fuck buddies

Booty calls</td><td>Super casual

Hook up Meet

One-night stand</td><td></td></tr>
<tr><td>Key factors differentiating partner types (from Figure 1)</td><td colspan="2">• Very high chance of reinfection
• Low chance of onward transmission to others
• High chance of expectations of sexual exclusivity
• Very high chance of sex again
• Enduring deep emotional connection
• Longer term relationship
• Highly contactable
• High likelihood of small assortative sexual mixing</td><td colspan="3">• High chance of reinfection
• Some chance of onward transmission
• High chance of sex again
• Developing emotional connection
• Developing time frame for the relationship
• Highly contactable
• High likelihood of small assortative sexual mixing</td><td colspan="2">• High chance of reinfection
• Some chance of onward transmission
• High chance of sex again
• Stable emotional connection
• Potentially enduring relationships
• Highly contactable
• Stable relationship
• Highly contactable</td><td>• Low chance of reinfection
• High chance of onward transmission
• Low perception of sexual exclusivity
• Low likelihood of sex again
• Little emotional connection
• Short or fleeting relationships
• Potentially contactable
• High likelihood of large disassortative sexual mixing within a large sexual network</td><td></td></tr>
<tr><td>Further explanatory notes</td><td colspan="2">• Could be primary partner – spouse/civil partner wife/husband
• Could be secondary partner-include someone's long term 'affair'
• High likelihood of stable relationship
• Regular sex
• Future orientation – will have a significant past
• Potential co-habiting</td><td colspan="3">• Almost always sex on more than one occasion
• Future orientation – may or may not have much of a past
• Growing emotional connection
• High likelihood of a labile relationship</td><td colspan="2">• Sex at least on more than one occasion
• Anticipation of sex again
• No relationship potential
• Sex intermittent/irregular
• Non-romantic emotional connection
• Sex for pleasure
• Between other kinds of partners
• Sporadic
• Sex with eyes
• Concurrent
• Some likelihood of stable relationships</td><td>• One occasion only
• No anticipation of sex again
• No past no future
• Little/no emotional connection
• Recreational Sex
• High likelihood of labile relationship</td><td>• Sex in return for money or services/ goods</td></tr>
</table>

Legend for Table S1: The first row of the table shows the condensed, five-type partner typology, with the second row illustrating how they relate to the 'original' eight partner types described in Figure 1. The third row summarises the spectrum of issues detailed in Figure 1.
